# Application of neural network adaptive filter method to simultaneous detection of polymetallic ions based on ultraviolet-visible spectroscopy

**DOI:** 10.3389/fchem.2024.1409527

**Published:** 2024-09-05

**Authors:** Bo Wu, Fengbo Zhou

**Affiliations:** ^1^ School of Mechanical and Energy Engineering, Shaoyang University, Shaoyang, China; ^2^ Hunan Province Key Laboratory of Southwest, Hunan Academician Workstation, School of Information Science and Engineering, Shaoyang University, Shaoyang, China

**Keywords:** neural network adaptive filter, signal processing, noise reduction, quantitative analysis, ultraviolet-visible spectroscopy

## Abstract

A novel neural network adaptive filter algorithm is proposed to address the challenge of weak spectral signals and low accuracy in micro-spectrometer detection. This algorithm bases on error backpropagation (BP) and least mean square (LMS), introduces an innovative BP neural network model incorporating instantaneous error function and error factor to optimize the learning process. It establishes a network relationship through the input signal, output signal, error and step factor of the adaptive filter, and defines a training optimization learning method for this relationship. To validate the effectiveness of the algorithm, experiments were conducted on simulated noisy signals and actual spectral signals. Results show that the algorithm effectively denoises signals, reduces noise interference, and enhances signal quality, the SNR of the proposed algorithm is 3–4 dB higher than that of the traditional algorithm. The experimental spectral results showed that the proposed neural network adaptive filter algorithm combined with partial least squares regression is suitable for simultaneous detection of copper and cobalt based on ultraviolet-visible spectroscopy, and has broad application prospects.

## 1 Introduction

In the hydrometallurgy of extracting zinc, the current primary method of detecting the concentration information of copper and cobalt impurity metal ions relies on manual offline analysis (Zabiszak et al., 2021; Zhou et al., 2020; Sikder et al., 2018; Deluca et al., 2023). This approach means that the setting of industrial parameters in the process of hydrometallurgy lacks scientific basis. The real-time responsiveness is poor, the detection steps are cumbersome, and there is a significant lag time (Fawzy et al., 2023). The micro-fiber spectrometer, due to its characteristics such as miniaturization, integration, and rapid detection speed, is suitable for the online detection of multi-component substances in industrial settings (Attia et al., 2018; Giriraj and Sivakkumar, 2017; Martins et al., 2017; Dehghannasiri et al., 2017). However, as the micro-spectrometer adopts a single-beam structure and a CCD detector, when detecting the concentration of multiple metal ions in high zinc solution, issues arise due to the fluctuation of the light source, instrument circuit noise and interference from the base zinc ions (Jin et al., 2024; Zhang D. Y. et al., 2021; Liu et al., 2017; Zou et al., 2018). These factors lead to weak spectral signals and poor accuracy, severely affecting the precision of spectral detection.

In actual detection, noise is ubiquitous, encompassing high-frequency noise, low-frequency noise, white noise, and various other types of noise signals. To mitigate the impact of these noise signals, they need to be removed (Zhou et al., 2019; Ford et al., 2018; Huang and Chen, 2021; Li and Zhao, 2023). Signal enhancement algorithms are mainly aimed at processing signals where the signal-to-noise ratio is low due to strong noise interference (Lee et al., 2015). How to reduce noise in signals has always been a focal research topic in signal processing. Presently, commonly used signal enhancement algorithms both domestically and internationally include wavelet signal enhancement algorithms, Savitzky-Golay denoising enhancement algorithms, and LMS algorithms (Chu et al., 2021; Huang et al., 2020). The LMS algorithm, due to its low computational complexity, rapid convergence and high stability, has been widely applied worldwide. However, through research on the LMS algorithm, it is found that it inherently has some flaws. For instance, the conventional LMS algorithm has a relatively slow convergence speed and requires a longer denoising time (Sibtain et al., 2022). Therefore, it is necessary to improve the convergence speed and make the algorithm stable in a shorter time. It is clear that while traditional methods provide accuracy, they lack real-time capability and efficiency. Advanced spectroscopic techniques offer speed and integration but are hindered by noise and interference issues. Existing signal enhancement algorithms each have their strengths and weaknesses, with the LMS algorithm being notable for its balance of simplicity and performance despite its slower convergence.

In this paper, a neural network adaptive filtering algorithm (NNAF) based on BP (backpropagation) and LMS (Least Mean Square) is studied to improve the accuracy and real-time performance of online detection of impurity metal ion concentration (Zhang and So, 2020; Liu et al., 2015; Zhang and Luo, 2023; Zheng et al., 2020; Shi et al., 2021). As a widely used learning mechanism in neural network, BP algorithm adjusts the weight by calculating the output error and propagating it back to the network (Mancini et al., 2021). LMS algorithm is an adaptive filtering algorithm based on gradient descent principle, which has good convergence and robustness. The proposed neural network adaptive filtering algorithm integrates adaptive filtering and neural network error compensation (LeCun et al., 2015; Zhang C. et al., 2021; Huang et al., 2023). It adopts a new BP neural network model, combining instantaneous error function and error factor to improve the learning process. The network relationship is established through the input signal, output signal, error and step factor of the adaptive filter, and the training optimization method suitable for this relationship is determined. The experimental results show that the neural network adaptive filtering algorithm (NNAF) shows superior denoising ability, effectively reduces noise interference and improves signal quality.

## 2 NNAF adaptive denoising algorithm

### 2.1 NNAF algorithm

**FIGURE 1:**
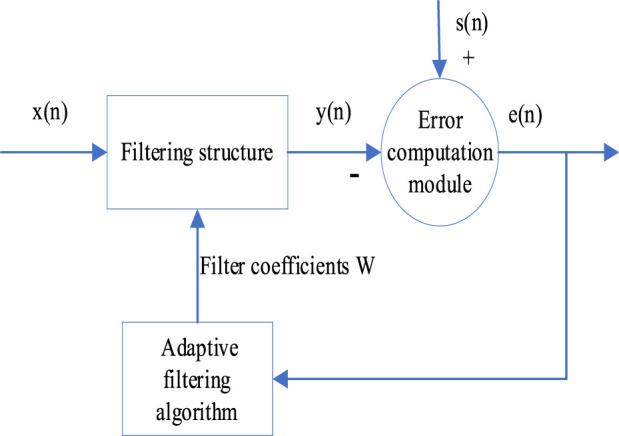
Schematic diagram of the LMS algorithm.

**FIGURE 2:**
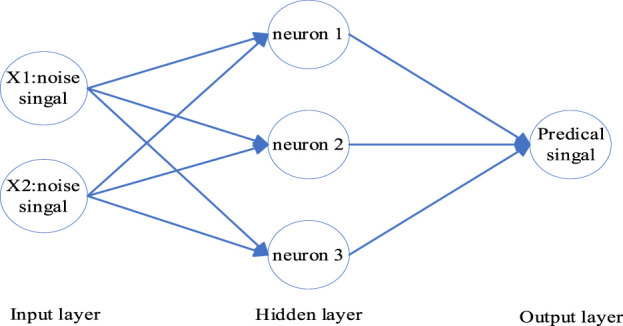
Diagram of the BP structure.

The LMS algorithm is an adaptive filtering method, extensively employed in the realm of signal processing and noise reduction. Despite its broad application, its convergence rate remains relatively slow. The schematic of the LMS principle is depicted in Figure 1. The backpropagation (BP) algorithm is an optimization method used within neural networks. It operates by calculating output errors and backpropagating them through the network for weight adjustment. The structure of the BP is illustrated in Figure 2. The BP algorithm boasts significant competence in addressing non-linear and intricate issues. However, during the early phases of training, weight adjustments might render the network’s output exceedingly sensitive. Noise at the inception can propagate throughout the entire network, resulting in unstable training outcomes. Therefore, by amalgamating the BP and LMS algorithms, a neural network adaptive filter method (NNAF) is proposed.

The NNAF algorithm leverages the noise reduction benefits of the LMS algorithm and the BP algorithm’s strength in optimizing complex non-linear problems, incorporating a new BP neural network model. During the backpropagation process, instantaneous error functions and error factors are introduced to optimize the learning procedure. The network relationship is established using the adaptive filter’s input signal, output signal, error, and step factor, and an optimized training procedure tailored to this relationship is identified. This algorithm successfully combines adaptive filtering with neural network error compensation. Through this approach, the NNAF algorithm effectively minimizes noise interference and enhances signal quality.

### 2.2 Implementation of the NNAF algorithm

**FIGURE 3:**
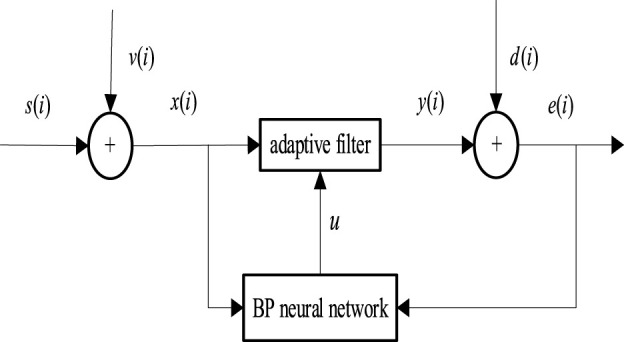
Schematic diagram of the NNAF algorithm.

The implementation of the NNAF adaptive denoising algorithm is primarily an efficient and intertwined process. This procedure integrates the strategies of LMS filtering and BP optimization, fully harnessing the strengths of both to enhance denoising performance. The schematic of this algorithm is depicted in Figure 3.

Where $x\left(i\right)$ and $e\left(i\right)$ are the input signals for the BP neural network, and u is the output of the neural network training results. The NNAF algorithm establishes a learning network structure, seeking the optimal learning step factor u through the error $e\left(n\right)$ and input signal $d\left(n\right)$ . By learning from given sample data, it establishes a real-time data correlation model. The rules for updating the parameters of the NNAF algorithm are Equation 1, Equation 2 and Equation 3.

$$e\left(n\right)=d\left(n\right)-X^{T}\left(n\right)W\left(n\right)$$ (1)

$$W\left(n+1\right)=W\left(n\right)+ue\left(n\right)X^{T}\left(n\right)$$ (2)

$$u=\left\{\begin{array}{l} u_{\max},\beta >u_{\max}\\ u_{\min},\beta <u_{\min}\\ \beta \end{array}\right.$$ (3)

To ensure the convergence of the neural network algorithm, β must satisfy $\beta <\frac{n}{\lambda_{\max}}$ (where $\lambda_{\max}$ is the maximum eigenvalue and is positive). Using $u_{\min}$ ensures that u is still influenced by the changes in the output signal and error. In practical applications, $u_{\max}$ and $u_{\min}$ can be determined experimentally. To speed up convergence, instantaneous error function y and error factor ee proposed by this algorithm are shown in Equation 4 and Equation 5.

$$y=b\left(-0.5+\frac{1}{1+e^{-a\left|x\right|}}\right)$$ (4)

In Equation 4, the steepness of the Sigmoid function is directly determined by parameter a , which is inversely related to the speed at which the function curve rises. b characterizes the range of values of the dependent variable in the Sigmoid function, determining the height of the curve.

$$ee=e^{x{|}^{r}}-1$$ (5)

In Equation 5, ee represents the error factor, and r represents the rate of exponential growth. It can also be understood as the rate at which the correction factor is adjusted. The larger r is, the greater the instantaneous error, and the greater the correction to the step factor. At this time, the step factor at the initial moment is larger, which further updates the required weight vector value. Through experimental simulation, the optimal value of parameter a is 5, and the optimal value of parameter r is 6., as shown in Equation 6:

$$u=\left(-0.5+\frac{1}{1+e^{-5\left|x\right|}}\right)\left(e^{{\left|x\right|}^{6}}-1\right)$$ (6)

**FIGURE 4:**
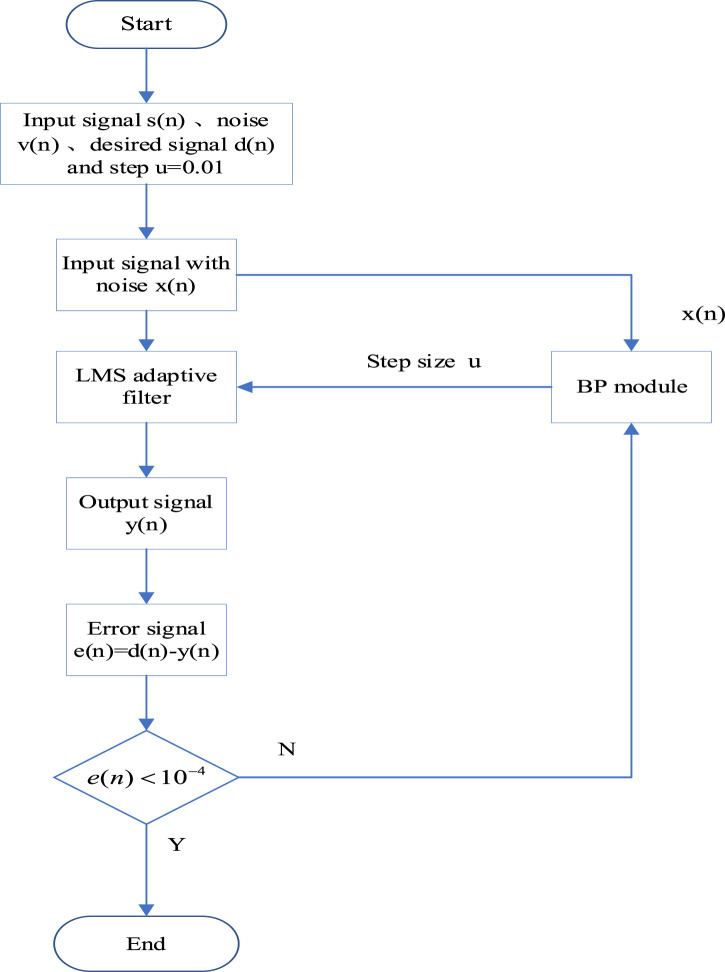
Flowchart of the NNAF algorithm.

The NNAF algorithm optimizes the learning process by incorporating an instantaneous error function y and error factor ee . It uses the input signal $x\left(n\right)$ , output signal $y\left(n\right)$ , error $e\left(n\right)$ , and step factor u of the adaptive filter to establish a network relationship and determines the training optimization learning steps for this relationship. The flowchart of the NNAF algorithm is shown in Figure 4. First, initialize the step vector u and process the input signal $x\left(n\right)$ with LMS filtering. Then, the error $e\left(n\right)$ between the target output and the filter output is backpropagated to BP. By calculating the gradient of the error and then updating the step factor in the negative direction of the gradient, the output of the filter becomes closer to the expected output. This also further reduces the error, achieving global optimization. Finally, the LMS algorithm and BP algorithm interactively feedback. When the error signal $e\left(n\right)$ is less than $10^{-4}$ , the iteration ends, and the filtered signal is output.

## 3 Experimental

### 3.1 Simulated data

**FIGURE 5:**
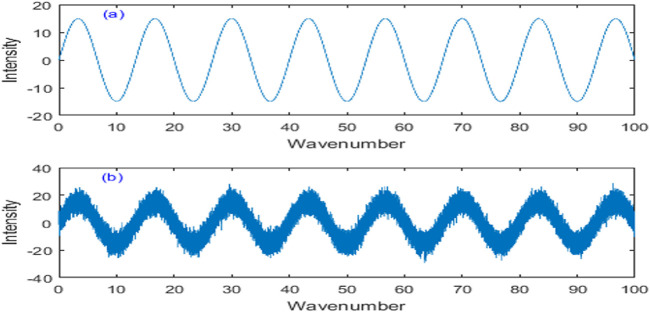
Simulated original signal and noisy signal. **(A)** Original signal. **(B)** Noisy signal.

The simulation data is used to select the adaptive step factor of the neural network adaptive filter algorithm (NNAF) and verify the denoising performance. The pure signal uses sine wave signal provided by MATLAB software, and then adds noise with signal-to-noise ratio (SNR) of 15 dB. The original signal and noisy signal are shown in Figure 5.

### 3.2 Experimental spectral signal

Add the standard solution of copper, cobalt and zinc, 7.5 ml of acetic acid-sodium acetate solution and 5.00 ml of nitroso R salt solution in turn into a 25 ml calibration flask and dilute with distilled water. Shake well to complete the reaction of the elements to be detected, and prepare a blank reagent reference in the same way. The solution to be tested and the reference solution were placed in a 1 cm cuvette and measured by the Optosky ATP2000 spectrophotometer in the wavelength range of 200–1,100 nm. The final concentration ranges were 0.5–5 mg/L for copper, and 0.3–3 mg/L for cobalt. All measured spectra were the average of 5 replicates.

## 4 Results and discussion

### 4.1 Comparison of convergence performance

In order to verify the effectiveness of NNAF algorithm, the performance of NNAF algorithm is compared with other commonly used algorithms. The comparison of the convergence error performance of the three algorithms is shown in Figure 5. The input signal is a noisy sine wave, and the simulation parameters are set as follows.

1) The order L of the filter is set 10. The initial weight $w\left(n\right)$ of the adaptive filter is defined as 0, and added noise $v\left(n\right)$ is a zero-mean independent Gaussian random sequence with a variance of 0.04.
2) For the fixed-step LMS algorithm, its learning step factor u is a small positive number, step factor u set to 0.008. For the variable step-size algorithm, its step size is variable. $u_{\max}$ and $u_{\min}$ are 0.009, 0.0006 in the algorithm respectively.
3) Average statistical time is 20, and the sample size is 1,000. The greed algorithm is used in the NNAF model to find training samples, with some data shown in Table 1.
4) A BP model is established, which includes an input vector with 10 components (assuming the input signal of the adaptive filter is 12 and the deviation is 2) and 25 hidden units. The model is trained through the neural network to scan the output results, thereby generating the optimal step size factor u .

**TABLE 1:** Partial training sample data of the BP model.

Input signal x(n)	Error	u
−1.1676 1.0718 1.1786 1.5808 -1.3713 -0.9793 -0.7802 1.7091 -0.0351 0.1680	0.3008	0.310
0.7880 0.7925 0.1972 -0.0696 -0.0867 -0.0277 -0.0048 0.0084 0.0157 -0.0065	0.6324	0.800
0.8307 0.8292 0.2088 -0.0964 -0.1078 -0.0334 0.0147 0.0242 0.0162 0.0068	−0.4133	0.5090
0.0070 0.0141 0.0212 0.0282 0.0352 0.0424 0.0494 0.0565 0.0636 0.0706	−0.2865	0.0037
0.7560 0.7489 0.7418 0.7348 0.6995 0.6924 0.6854 -1.9866-1.9364 -1.9601	0.0183	0.4396

**FIGURE 6:**
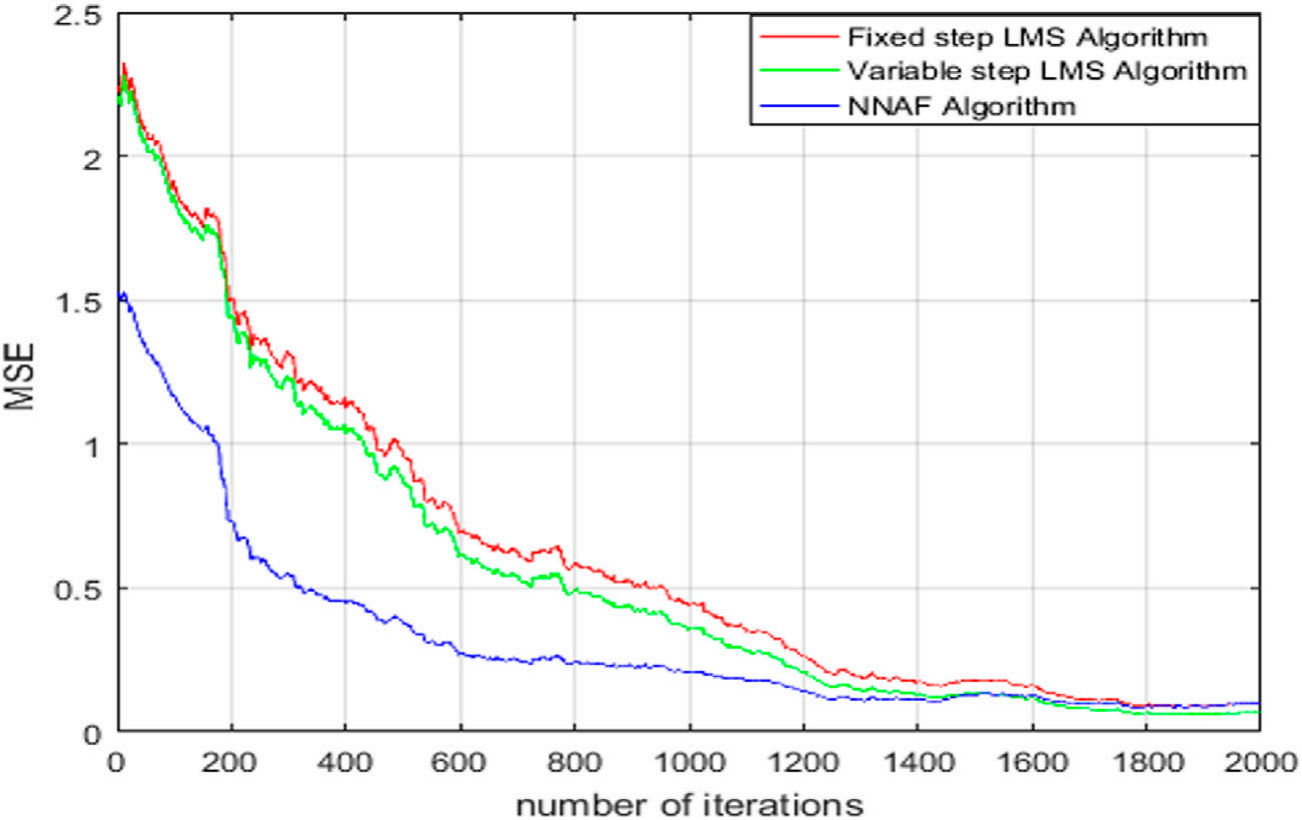
Error convergence curve diagram.

It can be observed from Figure 6 that the NNAF algorithm allows the adaptive filter to achieve higher convergence speed compared with the other three algorithms.

### 4.2 Noise elimination of simulation data

**FIGURE 7:**
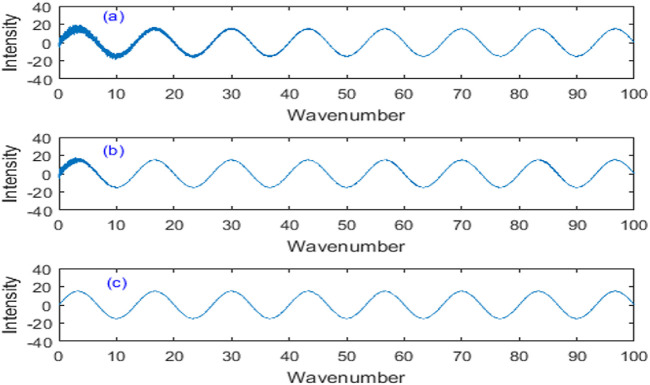
Comparison of denoising effects using different methods. **(A)** Fixed step LMS algorithm. **(B)** Variable step LMS algorithm. **(C)** NNAF algorithm.

The denoising effect of NNAF method was compared with that of other denoising methods. The comparison of denoising effects using different methods is shown in Figure 7. In Figure 7A, the denoised signal using fixed step LMS method retained a lot of noise, and the denoising effect was obviously poor. In Figure 7B, the denoised signal using variable step LMS method was relatively smooth, but the denoising effect at the beginning is not good. In Figure 7C, the denoised signal using the proposed NNAF method is smooth and retains the peak characteristics, which is in good agreement with the original signal, so it has good denoising performance.

In order to further verify the performance of the proposed NNAF method, different signal-to-noise ratios are added to the original signal. The signal-to-noise ratio (SNR) and root mean square error (RMSE) of denoised signals obtained by different methods are shown in Table 2. Compared with other methods, the denoised signal using NNAF method has the highest SNR and the smallest RMSE under different signal-to-noise ratios. Therefore, the simulation results strongly show that the proposed NNAF algorithm achieves superior denoising performance, which verifies its theoretical feasibility.

**TABLE 2:** The calculation results of the denoised signal by different methods.

Denoising methods	5 dB		15 dB		25 dB	
	SNR/dB	RMSE	SNR/dB	RMSE	SNR/dB	RMSE
Fixed step LMS algorithm	7.1391	0.5493	22.4684	0.5391	29.2929	0.3634
Variable step LMS algorithm	9.7945	0.3545	27.5210	0.2987	32.4876	0.2514
NNAF algorithm	16.7149	0.2945	28.2662	0.2514	36.3937	0.1603

### 4.3 Spectral processing

**FIGURE 8:**
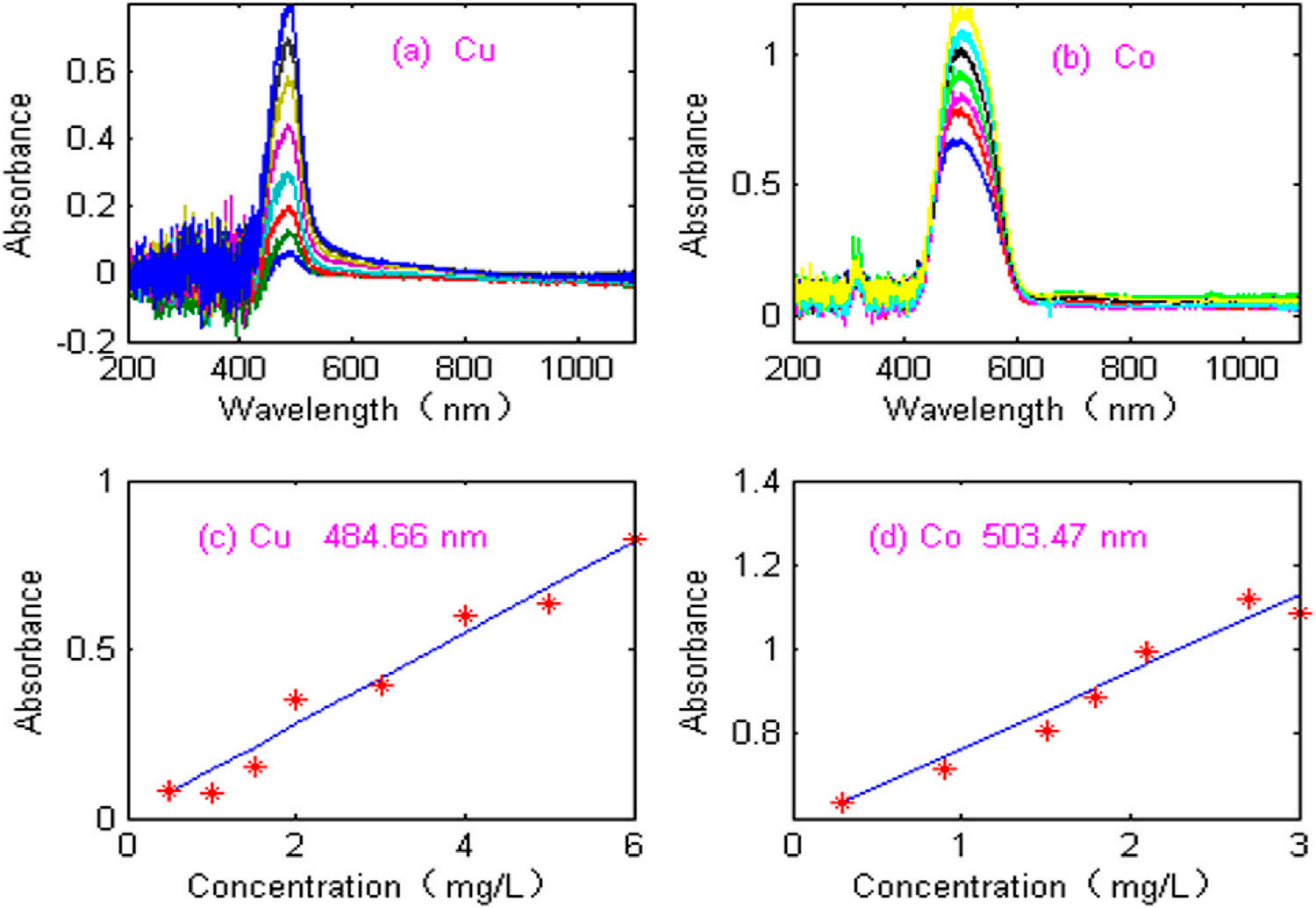
Acquisition of spectral signal. **(A)** Absorbance spectra of Cu. **(B)** Absorbance spectra of Co. **(C)** Calibration curves of copper. **(D)** Calibration curves of cobalt.

The proposed NNAF method was applied to the experimental ultraviolet-visible spectral signal. Figure 8A shows absorption spectra of copper (Cu) in the wavelength range of 200–1,100 nm, where the concentration of copper ranged from 0.5 to 6.0 mg/L. Figure 8B shows absorption spectra of cobalt (Co), where the concentration of cobalt ranged from 0.3 to 3.0 mg/L. As can be seen from Figure 8, the spectral signals of copper and cobalt are seriously disturbed by noise. The maximum absorbance of copper and cobalt are at the wavelengths of 484.66 nm and 503.47 nm, respectively. In order to evaluate the linearity, the calibration curves of copper and cobalt at the maximum absorbance were constructed. As can be seen from Figures 8C, D, the linearity of copper and cobalt is poor. The linear equation and linear coefficient of copper is: Abs = 0.1359 C_Cu_+ 0.0061 (R2 = 0.9908). The linear equation and linear coefficient of cobalt is Abs = 0.1834 C_Co_+ 0.5801 (R2 = 0.9926). Therefore, it is necessary to denoise the experimental spectrum and improve the detection accuracy.

**FIGURE 9:**
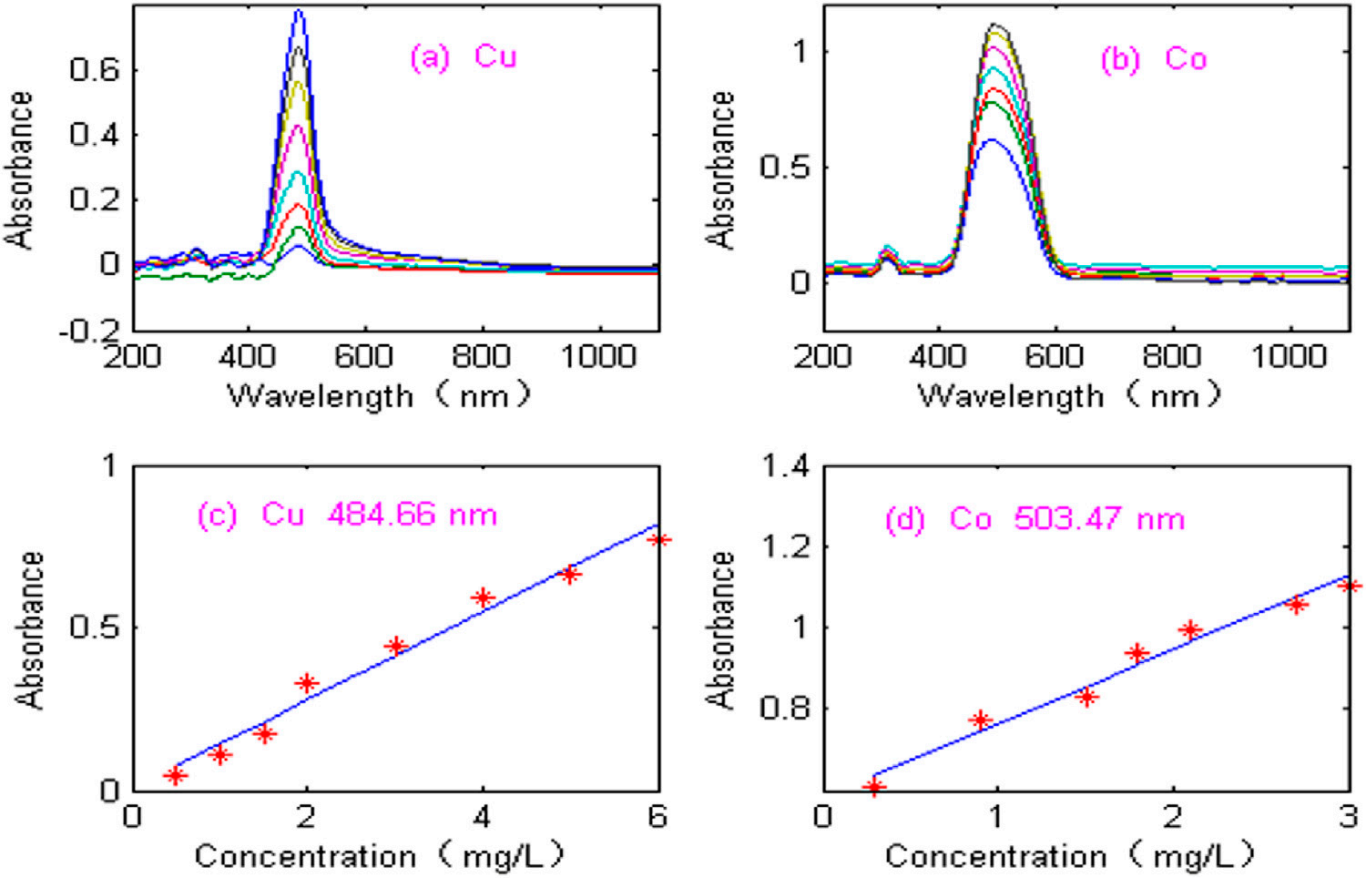
Spectral processed signal using NNAF method. **(A)** Absorbance spectra of Cu. **(B)** Absorbance spectra of Co. **(C)** Calibration curves of copper. **(D)** Calibration curves of cobalt.

The Optosky ATP2000 micro spectrometer has the advantages of intelligence, miniaturization, modularization and fast detection speed, but it adopts single beam structure and CCD detector, which leads to noise affecting the detection performance of spectrometer. Therefore, the spectral signal is disturbed by noise, the accuracy of simultaneous detection of copper and cobalt will be seriously affected if the spectral data is directly modeled without denoising pretreatment. The proposed NNAF method is used to process spectral signal and eliminate high-frequency and low-frequency noise. Figure 9A shows the denoising signal of copper. Figure 9B shows the denoising signal of cobalt. As can be seen from Figures 9A, B, the denoised signals of copper and cobalt are smooth, and the signal shape is consistent with the expectation. In order to evaluate the performance of NNAF method, Figures 9C, D show the calibration curves of the denoised copper and cobalt signals at the maximum absorbance. The correlation coefficients of Cu and Co are 0.9952 and 0.9967, respectively. The results show that the proposed NNAF method significantly improves the linear relationship between copper and cobalt, which is beneficial to improve the accuracy of simultaneous detection of copper and cobalt.

### 4.4 Simultaneous detection of copper and cobalt

In order to evaluate the performance of NNAF algorithm in experimental spectrum processing, we prepared 10 groups of mixed solutions containing different proportions of copper and cobalt, in which the zinc concentration was fixed at 20 g/L for reference. PLS (partial least square method) and NNAF-PLS (neural network adaptive filter algorithm combined with partial least square method) were used to simultaneously detect copper and cobalt. The root mean square error of prediction values (RMSEP) and average relative deviation (ARD) are used as evaluation indexes, and the predicted concentrations of cop-per and cobalt are shown in Table 3. It can be concluded from Table 3 that the prediction performance of NNAF–PLS method is far superior to that of PLS method. Using NNAF–PLS method to detect copper and cobalt simultaneously, the RMSEP for copper and cobalt were 0.104 and 0.048, respectively; the ARD of copper and cobalt were 3.342% and 2.521%, respectively, which meets industrial production indicators.

**TABLE 3:** The predicted results of copper and cobalt by PLS and NNAF–PLS methods.

No.	Actual value (mg/L)		Predicted value by PLS		Predicted value by NNAF–PLS	
	Cu	Co	Cu	Co	Cu	Co
1	2.0	1.5	1.902	1.563	1.963	1.519
2	3.0	1.2	3.284	1.302	2.884	1.241
3	5.0	2.7	5.425	2.535	5.106	2.668
4	4.0	0.6	4.361	0.641	4.145	0.612
5	1.5	3.0	1.417	3.212	1.436	3.109
6	3.5	0.3	3.287	0.322	3.397	0.311
7	4.5	0.9	4.922	0.944	4.703	0.931
8	1.0	2.4	0.934	2.142	0.962	2.345
9	2.5	2.1	2.773	2.251	2.556	2.145
10	0.5	1.8	0.531	1.907	0.521	1.839
The average relative deviation (%)			7.661	6.872	3.342	2.521
RMSEP			0.267	0.138	0.104	0.048

**FIGURE 10:**
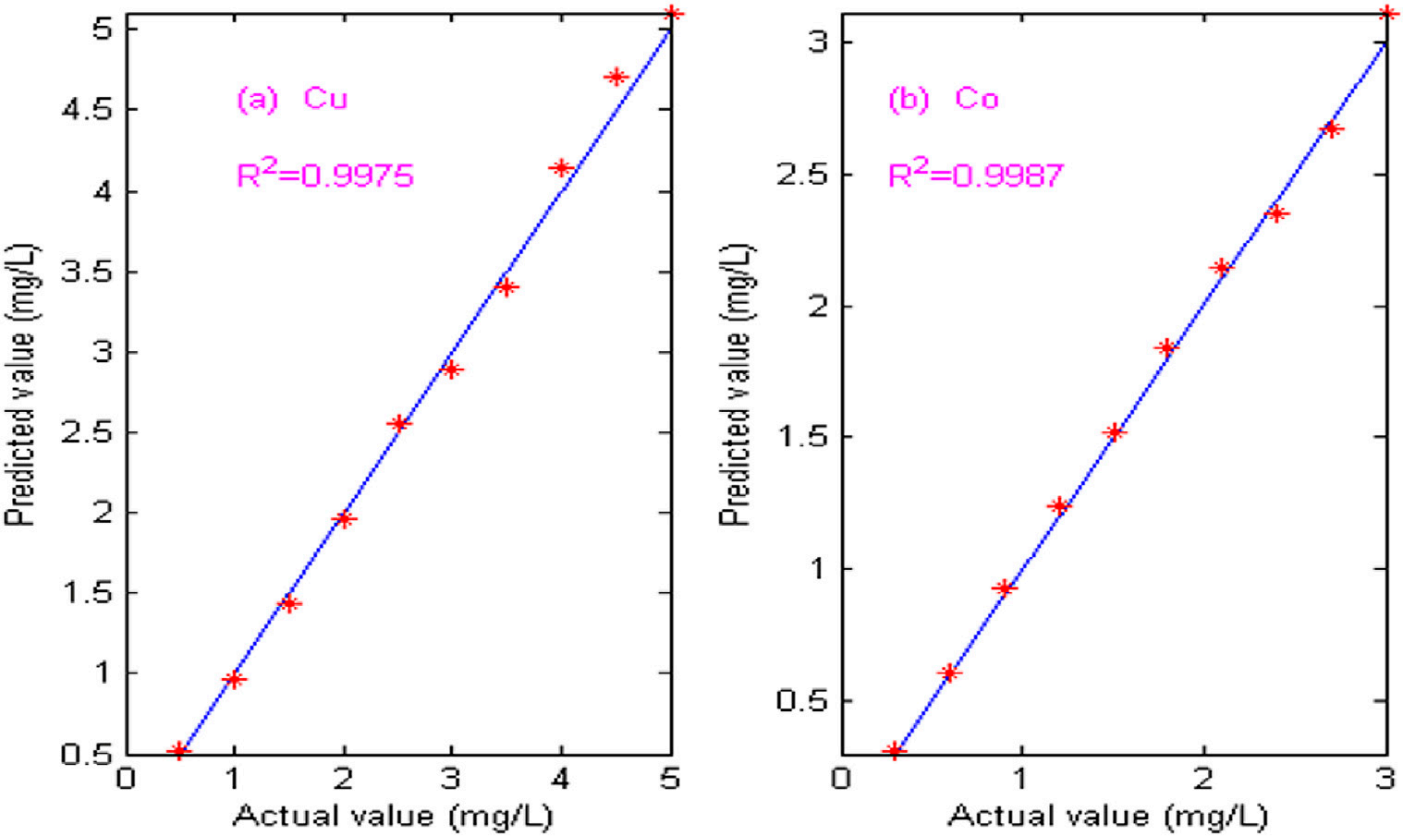
Predicted and actual values of copper and cobalt. **(A)** Copper. **(B)** Cobalt.

Figure 10 shows the calibration curve between the predicted value and the actual value of copper and cobalt. From Figure 10, it can be seen that the predicted values and the actual values of copper and cobalt are almost the same, and the correlation coefficient (R^2^) of copper is 0.9975 and that of cobalt is 0.9987. The experimental results show that this proposed NNAF method is suitable for on-line detection of copper and cobalt in zinc hydro-metallurgy, and has broad application prospects.

## 5 Conclusion

This paper presents a neural network adaptive filter algorithm (NNAF) based on error backpropagation (BP) and the least mean square (LMS). Through in-depth research and experimental processing of actual signals, we found that this algorithm can effectively reduce noise interference and improve signal quality, showing great potential for application. The algorithm fully leverages the advantages of both BP and LMS. It not only utilizes BP’s efficacy in error backpropagation but also incorporates the denoising characteristics of LMS, further enhancing the performance of the denoising algorithm. The experimental spectral results showed that the proposed neural network adaptive filter algorithm (NNAF) combined with partial least squares regression is suitable for simultaneous detection of copper and cobalt based on ultraviolet-visible spectroscopy. The work in this paper is an effective attempt to detect polymetallic ions online in the process of zinc hydrometallurgy, and the proposed neural network adaptive filter method is also suitable for other spectral signals, such as infrared spectra, Raman spectra, and more.

## Data Availability

The raw data supporting the conclusions of this article will be made available by the authors, without undue reservation.
